# Primary MSCs for Personalized Medicine: Ethical Challenges, Isolation and Biocompatibility Evaluation of 3D Electrospun and Printed Scaffolds

**DOI:** 10.3390/biomedicines10071563

**Published:** 2022-06-30

**Authors:** Andrei Marian Feier, Diana Portan, Doina Ramona Manu, Vassilis Kostopoulos, Athanasios Kotrotsos, Gabriela Strnad, Minodora Dobreanu, Andreea Salcudean, Tiberiu Bataga

**Affiliations:** 1 Doctoral School, George Emil Palade University of Medicine, Pharmacy, Science, and Technology of Targu Mures, 540142 Targu Mures, Romania; andrei.feier@umfst.ro; 2 Center for Advanced Medical and Pharmaceutical Research, George Emil Palade University of Medicine, Pharmacy, Science, and Technology of Targu Mures, 540142 Targu Mures, Romania; doinaramonamanu@gmail.com (D.R.M.); minodora.dobreanu@umfst.ro (M.D.); 3 Department of Mechanical Engineering and Aeronautics, University of Patras, Patras University Campus, 26504 Patras, Greece; kostopoulos@upatras.gr (V.K.); akotrotso@gmail.com (A.K.); 4 Faculty of Engineering and Information Technology, George Emil Palade University of Medicine, Pharmacy, Science, and Technology of Targu Mures, 540142 Targu Mures, Romania; gabriela.strnad@umfst.ro; 5 Department of Ethics and Social Sciences, George Emil Palade University of Medicine, Pharmacy, Science, and Technology of Targu Mures, 540142 Targu Mures, Romania; andreea.salcudean@umfst.ro; 6 Department of Orthopedics and Traumatology, George Emil Palade University of Medicine, Pharmacy, Science, and Technology of Targu Mures, 540142 Targu Mures, Romania; tbataga@gmail.com

**Keywords:** personalized medicine, biocompatibility, tissue engineering, novel scaffolds

## Abstract

Autologous cell therapy uses patients’ own cells to deliver precise and ideal treatment through a personalized medicine approach. Isolation of patients’ cells from residual tissue extracted during surgery involves specific planning and lab steps. In the present manuscript, a path from isolation to in vitro research with human mesenchymal stem cells (MSCs) obtained from residual bone tissues is described as performed by a medical unit in collaboration with a research center. Ethical issues have been addressed by formulating appropriate harvesting protocols according to European regulations. Samples were collected from 19 patients; 10 of them were viable and after processing resulted in MSCs. MSCs were further differentiated in osteoblasts to investigate the biocompatibility of several 3D scaffolds produced by electrospinning and 3D printing technologies; traditional orthopedic titanium and nanostructured titanium substrates were also tested. 3D printed scaffolds proved superior compared to other substrates, enabling significantly improved response in osteoblast cells, indicating that their biomimetic structure and properties make them suitable for synthetic tissue engineering. The present research is a proof of concept that describes the process of primary stem cells isolation for in vitro research and opens avenues for the development of personalized cell platforms in the case of patients with orthopedic trauma. The demonstration model has promising perspectives in personalized medicine practices.

## 1. Introduction

Human mesenchymal stem cells (MSCs) were firstly described in 1974 by Friedenstein and his colleagues who demonstrated their osteogenic and multiplying potential [1]. Two decades later, the same team explored MSCs in an in vivo performance demonstrating their usefulness in restoring and regenerating cartilaginous, bone and adipose tissue. They have been afterwards studied, used, and researched in hospital practices for more than three decades. Later, a group of researchers isolated the cells from a porcine intestine to obtain a collagen matrix [2]; this was then covered with muscle and fibroblasts to produce connective epidermal tissue. After this breakthrough, plastic scaffolds were implemented and were universally populated with stem cells [3]. Several clinical trials, together with animal models provided insight and novelty in the treatment and research protocols of autoimmune diseases, regenerative therapies, and tissue injury [4]. Nowadays, investigations on adult stem cells are considered an extremely active field that has a dynamic evolution sustained by permanent emergence of novel information [5,6]. Contemporary researchers describe a high attraction to use bone-derived mesenchymal stem cells (BDMSCs) and therefore primary cell cultures, tightening regulations and rules in this field, especially in America and Europe [7]. Hence, an issue is encountered regarding the ease of access in conducting preclinical experiments with BDMSCs that aim to obtain advanced and modern implants with improved biocompatibility features [8]. Due to increased comfort, commercial cell lines are generally employed in such studies that involve biological testing of implantable materials. Few advantages that support and promote the use of commercial cell lines in biocompatibility and biomaterials science are greater phenotypic stability, an unlimited number of cells, and ease of culture; it is presumed that these cells models do act like primary osteoblast cells [9]. Despite that, it has been also accepted that differences between commercial and primary osteoblasts will alter the outcomes of biomaterials testing to an extent. Czekanska et al. evaluated the proliferation and maturation potential of three osteoblast commercial lines vs primary human osteoblast cells. They checked their suitability as in vitro paradigms for biomaterials testing and described equity in cell proliferation and mineralization between primary cells and some types of cell lines. However, commercial cell lines cannot be considered suitable replacements for primary cells; primary cells give a more complex response and faster feedback to the in vitro environmental stimuli [10].

One key point that must be referred before BDMSCs can be utilized for clinical therapy in human beings is the ethical safety aspect [11,12]. Commercial cell lines have unlimited availability and there are no safety issues associated to them [13,14]. However, numerous studies confirmed that between all types of MSCs, human bone-derived osteoblasts are preferable because they retain their osteogenic phenotype over time and over multiple passages, delivering an optimal source for both regenerative medicine and biocompatibility research [15,16,17].

All primary cell sources are part of the effort to provide more information on how cells behave, multiply, and react to the dysfunction that causes the disease to occur and how the early stages of human development unfold. Any such studies will ultimately lead to the future development of safe, effective, and novel therapies. One main subject of the present investigation is related to the isolation of human BDMCSs and their use for in vitro biocompatibility testing of 3D scaffolds. Encountered aspects related to deontology and ethics associated to this study are discussed below.

### 1.1. Ethical Compliance for MSCs

Research that involves human stem cells has been performed under constant controversy worldwide, particularly in the case of human embryonic stem cells. Human stem cells sources are divided in three categories: MSCs from fetal and adult tissues, human embryonic stem cells (hESCs), and human induced pluripotent stem cells (hiPSCs) respectively, each rising ethical aspect. MSCs obtained from fetal tissue (placenta, amnion fluid, cord blood, umbilical cord) or adult tissue (bone marrow, dental pulp, adipose tissue) share many characteristics and generally fulfil accepted criteria, such as: plastic adherence, certain surface marker expression, and an ability to differentiate into mesenchymal tissues [18]. Some ethical issues that all researchers face during clinical translation address risks of harm and potential benefits to society; the intention is towards minimizing risks of harm, selecting, and recruiting appropriate subjects, facilitating informed decision making through the consent form and process, and avoiding therapeutic misconception [19].

The general principles of creating, patenting, and using the above-mentioned stem cells categories are regulated at the European Council level through quality and safety standards for donation, obtaining, testing, processing, preservation, storage and distribution of human tissues and cells. Before any type of cell research is performed, informed consent should be obtained from a somatic cell donor. Informed consent is a major ethical concern in derivation and applications of human derived MSCs (HDSCs). Regarding the donation of human cells or tissues, principles such as the anonymity of both donor and recipient, altruism of the donor and solidarity between donor and recipient should be respected. As a matter of principle, human cells or tissues contained in advanced therapy medicinal products should be acquired from voluntary and unpaid donation. Member States of European Union are urged to take all necessary steps to encourage a strong public and nonprofit sector involvement to obtain human cells or tissues as voluntary and unpaid cell and tissue donations may contribute to high safety standards for cells and tissues and therefore to the protection of human health [20,21].

By now, 17 European countries have published clear regulations describing the policies that are involved in the field of stem cells research [22]. European countries’ position on human stem cell research can be grouped into five broad categories: very permissive, permissive with restrictions, restrictive by default, very restrictive, and unlegislated. Sixty-three percent of the countries studied fall into the first two categories. Romania, where the present research has been performed, is considered ‘restrictive by default’ as legislation is not explicit and national practices are restrictive [23]. In Europe, research and innovation in regenerative medicine are supported by legislation such as the so-called Biopatent Directive [24]. The research on MSCs and hiPSC is associated to less ethical and legal constraints than hESCs research and thus continues to make progress.

The ethical discussions have led to the development of multiple policies and rules adopted at the European level and some complementary rules applied by local committees. In all European countries, research projects that involve the use of HDSCs are reviewed by local ethics committees established in hospitals, research centers and higher education institutions such as faculties or universities. In order to guarantee that studies and projects are ethically and scientifically sound, research proposals go through a double process of verification both at the national and the European level and are submitted to the attention of a regulatory committee. The European Group for Ethics in Science and New Technologies is the regulatory institute issuing opinions on human-derived stem cells use.

The procedure of harvesting and subsequent isolation of osteoblasts derived from MSCs cannot be performed at a uni-disciplinary level; a multidisciplinary approach with the involvement of several hierarchical levels is required. The initial step is to acquire a favorable response from different ethics committees. A party capable of understanding and describing moral and political implications of stem cell research is compulsory. Further on, training sessions and workshops for all staff hierarchies involved in the study should be commenced [25]. Informed consent and voluntary enrolment approval are to be attained from participants. Depending on the research type, throughout the phase of donors’ enrolling, certain inclusion and exclusion criteria should be defined. Finally, harvesting of residual bone tissue implies contribution and compliance from an orthopedic surgeon; procedures pursuing isolation require the aid of a cell biology specialist; a laboratory technician is required for cell growth maintenance and equipment management. The proof of concept to demonstrate such a model was a goal of the present research.

### 1.2. Primary MSCs Differentiation and Biocompatibility Testing of 3D Scaffolds

MSCs are a heterogeneous set of cell populations that are defined by their potential to differentiate into a large series of mesenchymal lineage tissues, such as adipocytes, osteocytes, and chondrocytes. They also can differentiate in tissues that are not mesenchyme related (endothelial, neural, cardiac) and other cells [26]. The most common method to differentiate MSCs into different cell types is through their medium. There are many types of media that can be utilized for growing and isolating MSCs [27]. An important component of MSC media is the fetal bovine serum (FBS). It includes platelet-derived growth factor (PDGF), fibroblast growth factor and epidermal growth factor [28]. There is no gold standard reference regarding the usual culture media used for MSCs (alpha-MEM and DMEM). MSCs cultured on DMEM medium have low metabolic activity, without osteoblastic differentiation at the second passage of cell subcultures. Moreover, cell proliferation and growth are notably higher in the α-MEM environment. However, this was noticed only in the first seven days of culture, an event linked to the surplus of nutrients of this environment. Bone MSCs can differentiate into osteoblasts by addition of dexamethasone in their medium [29] or using innovative substrates that can program differentiation [30]; osteoblasts coming from MSCs can be also differentiated into adipocyte cells [31] chondrocytes [32] or neural cells [33,34]. Cell differentiation and functionality is lower with passage number, so early passage stem cells should be utilized in cell-based treatments [35,36]. At increased passage number, cells experience adaptations in morphology, reactions to stimuli, growth rates, protein expression and transfection performance, in comparison with the cells in earlier passages. Cell quality is of great importance to successfully achieve optimal end-results. Cells that were cultured for too long should be avoided to ensure well founded and reproducible results.

Biocompatibility studies offer reliable information with respect to the optimum required properties of newly designed materials for implant manufacturing. Two of the innovative technologies used to manufacture scaffolds for tissue engineering are electrospinning and 3D printing [37], which allow the programming of a design that involves complex geometries bound with internal and external selected features [38]. 3D scaffolds can mimic the properties of natural tissues and provide a template for cell development while stimulating tissue formation in vitro.

Nowadays we use a high number of manufacturing technologies and materials to produce biocompatible innovative scaffolds; the selection step to find the most appropriate structure for a specific application is essential. Efficient selection must be applied to reduce trial-and-error experimentation that is limited by high costs. Specific cell biomarkers are evaluated after cells deposition on the tested materials. Osteoblasts derived from MSCs are often used to evaluate the performance of novel 3D scaffolds with perspectives in bone tissue engineering. Basic parameters that give information associated to osteoblasts response to substrates are alkaline phosphatase (ALP) and total protein (TP). Their levels are decisive within the first hours after seeding on substrates. ALP is an enzyme found in bones with a key role in hard tissue formation. It is widely recognized as a biochemical marker for osteoblast metabolic activity. ALP levels in vitro indicate the degree of osteoblasts maturation, differentiation, and in vitro mineralization [39]. TP is also a known marker of osteoblast phenotype and plays a fundamental role for the quality of osteoblasts adhesion on substrates [40]. ALP per TP offers important information regarding a substrate biocompatibility and gives a more realistic image on what happens, compared to microscopy imaging. More precisely, they show the extent of an induced feedback in cells response and indicate the internal condition of cells as a function of a specific varying parameter in their external environment e.g., (substrate topography).

## 2. Materials and Methods

### 2.1. Tissue Harvesting and Lab Practices

Ethical approval to perform residual tissue extraction has been given by the Ethical Committee of the Mureș County Clinical Hospital (no. 4784 of 14 March 2017) and ‘George Emil Palade’ University of Medicine, Pharmacy, Science, and Technology of Târgu-Mureș (no. 158 of 23 June 2017), Romania. Patients involved were scheduled for total hip arthroplasty with cemented or uncemented total endoprostheses. Each patient has been informed previously to surgery, with respect to the purpose of using subsequent isolated cells for basic research studies and agreed to sign the consent. More precisely, they were informed about the use of residual bone tissue (i.e., spongy bone tissue from the femoral head) to conduct preliminary in vitro biocompatibility studies with the final goal to improve modern orthopedic implants.

**Figure 1:**
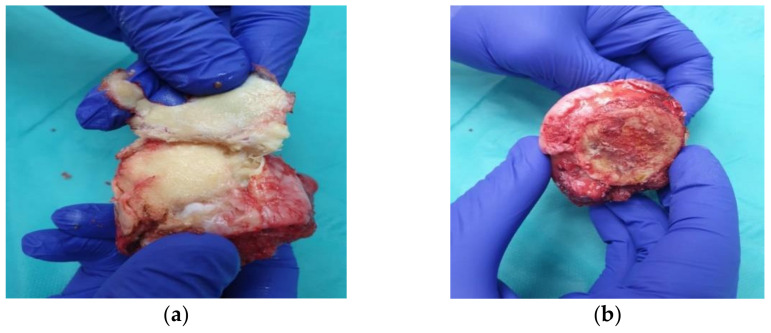
(**a**) Cartilage denudation and necrosis of the femoral head-macroscopic appearance and (**b**) Femoral head osteotomy site-thermo-generated necrosis of adjacent bone tissue.

For patient’s enrollment, inclusion criteria were established. Between the 72 patients who underwent inclusion screening, only 19 met the pre-established criteria. Included patients had a mean age of 60 years (SD ± 2.4) and 68% (n = 13) of subjects were female. All patients came from rural areas. Inclusion criteria with respect to the disease were defined as follows: patients diagnosed with primary hip osteoarthritis, rapidly evolving primary hip osteoarthritis, hip osteoarthritis secondary to congenital hip dysplasia, and post-traumatic hip osteoarthritis. Exclusion criteria were patients diagnosed with avascular necrosis of the femoral head (Figure 1), severe hormonal imbalances (hypothyroidism, hyperthyroidism, hepatomegaly, acromegaly, Hashimoto’s disease, Cushing’s syndrome, Graves’ disease) fatty liver disease, COVID-19 positive PCR test in the previous 8 weeks, any type of intra-articular injection treatment in the last 12 months, and treatment with any category of systemic corticosteroid compounds in the previous 12 months.

**Figure 2:**
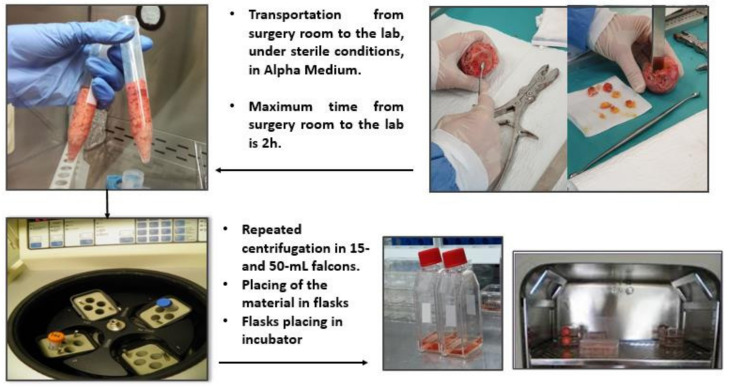
The schematic representation of steps involved in cells isolation: bone sectioning (up-right), bone deposition in 15 mL tubes (up-left), bone centrifugation (down-left) and medium with residual bone and extracted cells in flasks and in the incubator (down-right).

Residual tissue pieces have been extracted from the medullar area of the femoral head since it has been observed that these generate a higher number and improved quality of cell populations. An orthopedic curette and chisels were used to extract the tissue parts varying between 0.3 to 0.5 cm; further on, these were transferred to sterile plastic containers with alpha-MEM, 10% FBS and 1% penicillin/streptomycin. The sterile containers were then placed in a thermo-insolating box (Thermobox, Delta T, Fernwald, Germany) with ice and transported to laboratory in an interval of maximum 2 h. Bone fragments were washed with PBS to detach cell debris, erythrocytes and remaining fats and transferred in culture medium for MSCs. Centrifugation was applied for 8 min at an angular frequency of 2500 r/min, to facilitate the mobilization of MSCs from the stroma. Further on, the lipid layer was removed under laminar flow cabinet. Remaining medium with extracted cells and bone pieces were placed in T75 cell culture flasks. Each T75 flask contained about 5–6 pieces of bone and culture medium specific for MSCs: Alpha Minimal Essential Medium (Sigma Aldrich M4655, St. Louis, MO, USA) with 10% Fetal Bovine Serum (Sigma Aldrich F7524, St. Louis, MO, USA), 50 µg/mL Amphotericin B (Sigma Aldrich A2942, St. Louis, MO, USA), 25µg/mL Gentamycin (Sigma Aldrich G1397, St. Louis, MO, USA), 50 μg/mL L-ascorbic acid (Sigma Aldrich A4403, St. Louis, MO, USA). For osteogenic differentiation 10^−4^ M Dexamethasone (Sigma Aldrich D4902, St. Louis, MO, USA) has been added at the first passage. The schematic representation of the harvesting procedure may be seen in Figure 2.

Flasks were transferred to an incubator and were periodically verified using optic microscopy to eliminate those affected by possible microbiological contamination. Bone pieces were cleaned out with PBS after 4–5 days of incubation and the medium was replaced with a fresh one every 4–5 days.

Cells morphology has been periodically investigated after fixation with glutaraldehyde. Cells of 1st to 3rd passages were trypsinized and re-seeded in 24 well plates for seven and ten days in numbers of 3 × 10^4^, 6 × 10^4^ and 10^5^ cells/cm^2^. A very low amount of cells suspension, as calculated, has been placed in the middle of each well. After deposition, the well plates were left in an incubator for five hours to determine cells morphology. Culture medium has been added to cover the whole area of each well. Seven and ten days after incubation, cells were fixed by immersion in a 2.5% *w/v* glutaraldehyde solution in PBS for 60 min. Observations were made in phase contrast with an inverted Leica DMi8 microscope. 

### 2.2. Biomarker Analysis

The response of osteoblasts derived from primary MSCs to 3D scaffolds and to traditional orthopedic materials (standard orthopedic titanium) was investigated by analyzing ALP and TP levels in cells incubated on substrates for 1 and 3 days and by confocal imaging. One material with superior mechanical properties (PEI&HAp, PLA 60 μm) has been selected in each group category and tested for 7 days of culture. Surgeons, biologists, and engineers cooperated to perform the evaluation of several substrates with cells coming from patient’s own tissue. This research aimed to stimulate a cycle that will allow delivery of synthetic biocompatible platforms containing the patient’s own cells. Three main classes of substrates were selected to be tested: traditional orthopedic materials, 3D electrospinning scaffolds and 3D printing scaffolds. The description of the manufacturing methods for the tested substrates has been made in previous publications: titanium dioxide nanotubes (TNTs) were manufactured as described in [41]; electrospinning scaffolds were prepared according to the methodology described in previous papers and mechanically characterized [42,43]; 3D printed scaffolds were prepared as in [44] and were mechanically characterized. Cells were differentiated into osteoblasts by appropriate cell culture medium, as described in [45]. One to three passage cells were used for the investigation. During multiplication, proliferation and while seeded on the control material-tissue culture polystyrene (TCP), osteoblasts cultures were observed with an inverted Nikon Diaphot optical microscope. Cells populations containing ~6 × 10^4^ osteoblasts were seeded on each tested substrate. This number of cells has been selected based on a previous study where the seeding procedure is also described [46]. Cells were stained with Osteocalcin Monoclonal Antibody (Invitrogen OC4–30, Waltham, MA, USA) and ALPL Recombinant Rabbit Monoclonal Antibody (Invitrogen 7H11L3, Waltham, MA, USA) with Alexa Fluor 488 Goat Anti-Rabbit IgG (Invitrogen H+L, Waltham, MA, USA) and Alexa Fluor 594 Goat Anti-Mouse IgG (Invitrogen H+L, Waltham, MA, USA) as secondary antibodies, to observe their osteogenic differentiation. Cell viability data was analyzed with Viability/Cytotoxicity Assay Kit for Live & Dead Cells (Biotium 30002-T, Fremont, CA, USA) on every substrate tested. The ALP and the TP levels were measured after 1, 3 or 7 days of incubation on the substrates. The ALP enzyme activity was assessed with the P0757L kit from BioLabs (Frankfurt am Main, Germany). Bradford Protein Quantification kit (Bertin Pharma, Montigny-le-Bretonneux, France) was used to quantify TP level. An Infinite F200PRO UV/visible Spectrometer has been used to detect the ALP and the TP levels at 405 nm and 600 nm wavelengths, respectively. Experimental data were analyzed using SPSS 14.0 software (SPSS, USA). Cells were stained on substrates with an Actin Cytoskeleton/Focal Adhesion Staining Kit (MiliporeSigma FAK100, Burlington, MA, USA). Goat anti-mouse IgG antibody, FITC conjugated (Sigma Aldrich AP124F, St. Louis, MO, USA) was used as a secondary antibody. Confocal images were acquired (Leica True Confocal System SP8, Software: LAS X) to compare cells spreading and morphology on the different substrates.

## 3. Results

### 3.1. Osteoblasts Populations

**Figure 3:**
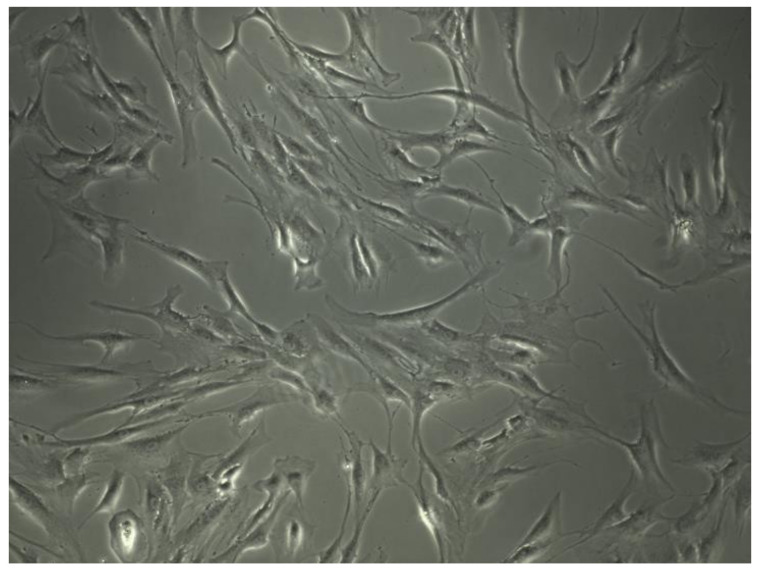
Human mesenchymal stem cells morphology under 10× optic microscopy.

Cells morphology is extremely important to evaluate cells physiologic condition. Human MSCs can be assigned to at least three morphological subpopulations: rapidly self-renewing cells; elongated, fibroblastic-like, spindle-shaped cells and slowly replicating, large, cuboidal or flattened cells (Figure 3).

**Figure 4:**
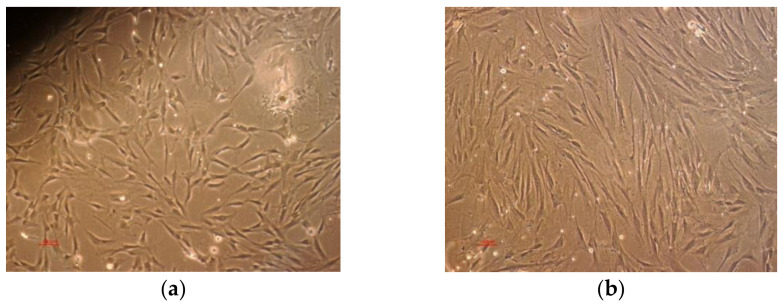
Optical microscopy (scale bar: 100 µm) images of: (**a**) Osteoblasts precursors at 10 days of incubation and (**b**) Osteoblasts after first passage.

**Figure 5:**
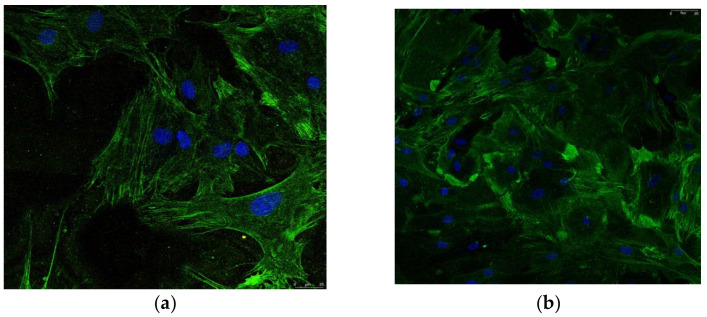
Confocal marker identification images of: Osteoblasts stained for (**a**) alkaline phosphatase and (**b**) osteocalcin.

Furthermore, distinct subpopulations could be associated with intrinsic qualities. Small cells manifest the highest multipotentiality. Wider, spindle-shaped cells show the greatest potential to differentiate into cartilage and larger MSCs are referred to as more mature that, in part, had differentiated into osteoblastic precursors [47]. After 7–10 days of incubation, the attached cells resulted and were observed on the flasks bottom (Figure 4a). After 4–6 weeks of incubation, a cell confluence of 85–90% was reached and the first passage of cells was performed (Figure 4b). The cells were spindle-shaped and large enough indicating a degree of differentiation. Differentiation was further evaluated by osteocalcin and alkaline posphatase staining; differentiated cells can be seen in Figure 5a,b.

### 3.2. Biocompatibility of Electrospun Subtrates

**Figure 6:**
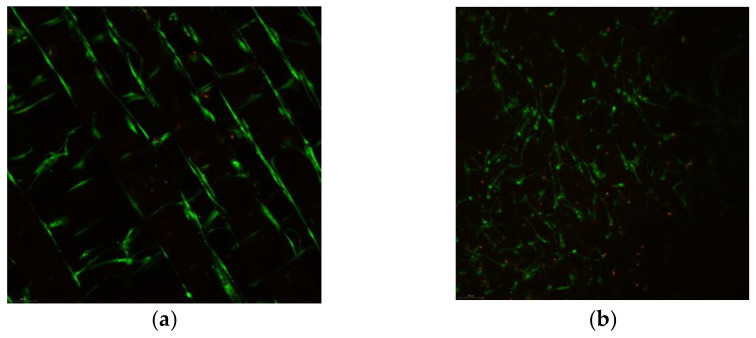
Osteoblasts cell viability tests on (**a**) 3D printed materials and (**b**) electrospun substrate.

Cell viability and cytotoxicity images can be observed in Figure 6. Osteoblasts show enhanced morphology on the tested substrates, with few death cells.

**Figure 7:**
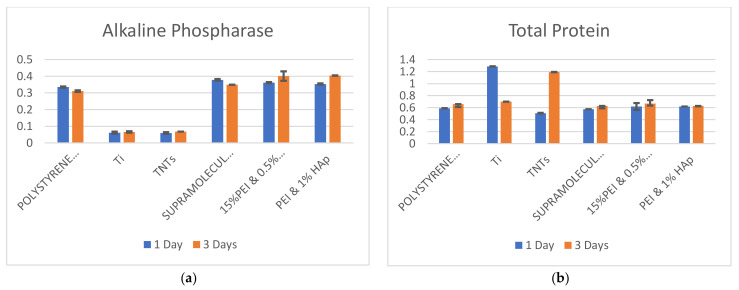

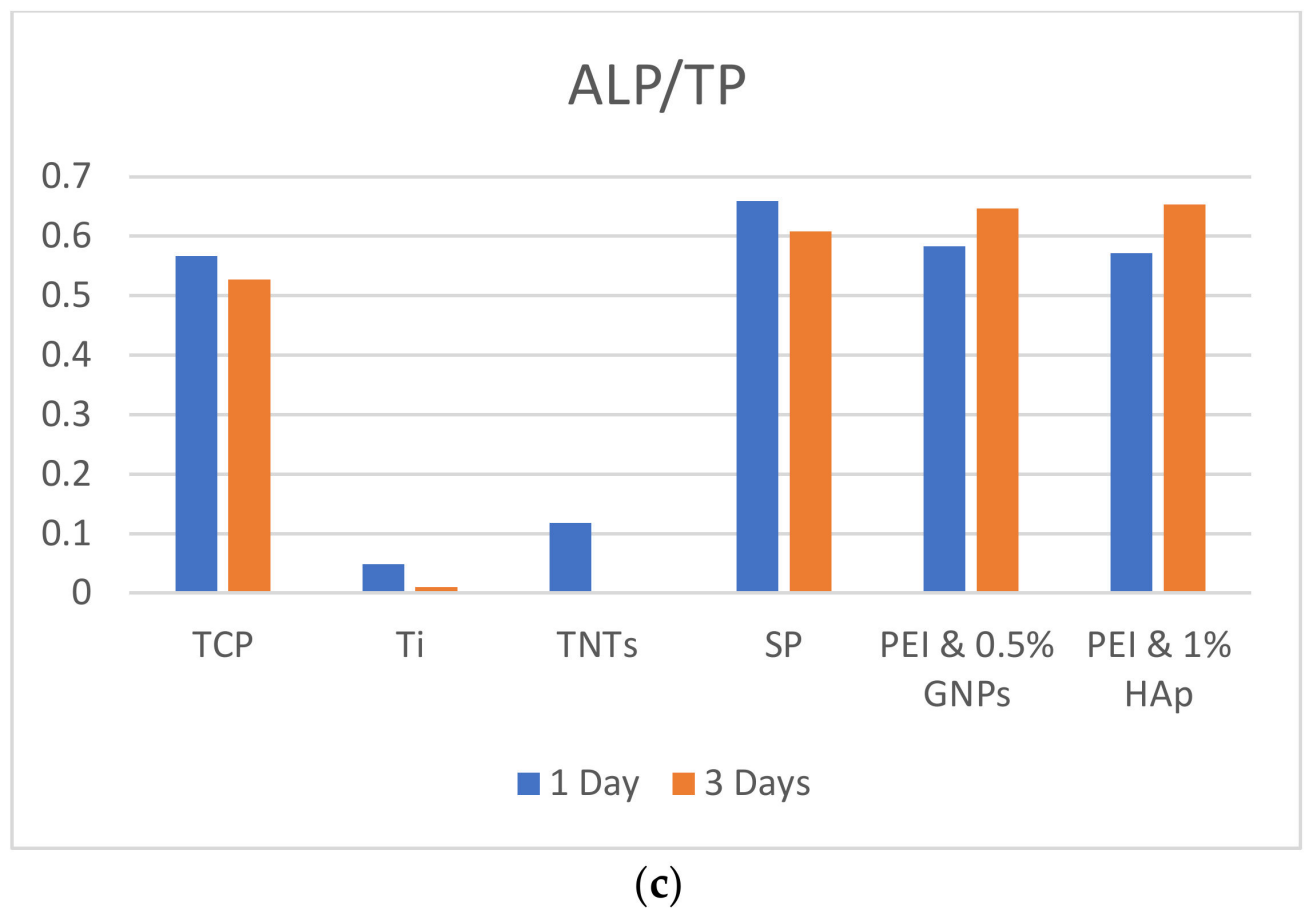
Levels of (**a**) ALP; (**b**) TP and (**c**) ALP/TP-in osteoblasts after 1- and 3-days incubation on polystyrene, titanium and electrospun substrates.

The diagrams in Figure 7 show the levels of ALP, TP, and their ratio in osteoblasts on TCP, pure titanium, TNTs and electrospun materials. Comparing the substrates, we observe a decreased ALP value in cells on TCP compared to SP and graphene nanoplatelets (GNPs)/hydroxyapatite (HAp) reinforced polyetherimide (PEI) (Figure 7a); the reason is that the electrospun fibers confer an extended contact surface through their nano-scale dimensions. This was expected and was already reported several times. It has been found that the electrospun materials are biocompatible and that they promote cells differentiation [48]; moreover, PEI has been declared biocompatible in vivo [47]. However, biocompatibility of SP hasn’t been previously evaluated. This material is mainly used in aeronautics. Due to its ease of manufacturing, as well as outstanding mechanical performance, it may find applications in orthopedic implantology.

As observed below, ALP quantity in osteoblasts on SP is lightly higher than in osteoblasts on TCP. However, it can be noticed that after 3 days of incubation, this quantity is reduced. ALP level is almost similar in osteoblasts populations on GNPs reinforced PEI and HAp reinforced PEI, for both 1 and 3 days of incubation. This indicates the reproducibility of the results since in both cases, cells come in contact with the PEI matrix and not with the reinforcement. The quantity of ALP increases in the third day of incubation. This indicates that both types of composites assure an appropriate microenvironment for osteoblasts development. At this point it may be affirmed that selection of one of the two electrospun composites for further investigations can be made according to their mechanical performance. A combination of GNPs and HAp reinforced PEI may be considered as well. Since higher ALP values will assure higher ALP/TP ratios, GNPs reinforced PEI and HAp reinforced PEI are superior to the other plastic substrates. It may be further observed in Figure 7b that TP values in osteoblasts on all substrates increase after 3 days of incubation, compared to day one. This is expected and is related to the bonds and adhesion of cells on substrates; connection is stronger as hours pass. Osteoblasts’ adaptation and adhesion to a substrate is crucial withing the first 72 h of cells incubation on the substrates; this process decides the material fate and the osseointegration rate. Alves and Wassall [49] reported interesting results after immersing implant samples into an osteoblast-like cell suspension for a period of 24, 48 and 72 h. After seeding the cells, samples were prepared for analysis with scanning electron microscopy. Based on surface analysis, osteoblastic cells adhered to the tested metallic surface after 24 h in culture. In 48 h, cells spread over the implant surface, and after 72 h a proliferation of cells with large and flat bodies was observed over the machined implant surface. Although both chemistry and surface structure of metallic and electrospun materials differ, the time of cells in vitro development can be similar for various substrates; despite that, cells’ morphology can be confusing and should not by itself be considered a key biocompatibility indicator. A real difference in the biocompatibility of substrates, which reflects the true change in cells’ internal state, may be assessed by analyzing the ALP/TP ratio.

The diagram in Figure 7c shows the ALP/TP ratios in osteoblasts on different categories of substrates. As observed, differences between the plastic substrates are not substantial. In the case of TCP and SP, the ALP/TP ratios are lower in day 1 compared to day 3; for GNPs reinforced PEI and HAp reinforced PEI the opposite happens.

Metallic substrates induce completely different feedback in cells compared to plastic scaffolds. Several studies [47,49] have previously demonstrated that strong adhesion of osteoblasts on titanium surfaces limits their movements, which negatively impacts their development. The interaction between titanium and osteoblasts is of two types, biochemical and electrostatic, respectively, while adhesion depends in a high extent on material topography and contact area. In Figure 7a, one may see that the ALP in osteoblasts on titanium is appreciable lower than on polymeric substrates. In Figure 7b, the TP level in cells on titanium substrates indicates the strong adhesion and the bond with the pure titanium/TNTs, by an increased protein amount in cells. Due to the high value of TP in the case of these two substrates, it is expected that the ALP/TP ratios will decrease, which can be clearly seen in Figure 7c. Furthermore, we observe (Figure 7b) that after 3 days of incubation on TNTs, the number of proteins is extremely high compared to day 1; the increased contact area conferred by the nano-dimension of the nanotubes leads to cells swallowing into the substrate; this has been previously proved [50]. Finally, Figure 7c shows that ALP/TP levels in cells on titanium and TNTs is extremely low compared to the polymeric materials. After 3 days of cell incubation on TNTs, this value cannot be distinguished in the diagram. Results underline that polymer-based composites such as GNPs reinforced PEI and HAp reinforced PEI enhance cellular activity after 3 days of incubation compared to metals.

### 3.3. Biocompatibility of 3D Printed Scaffolds

**Figure 8:**
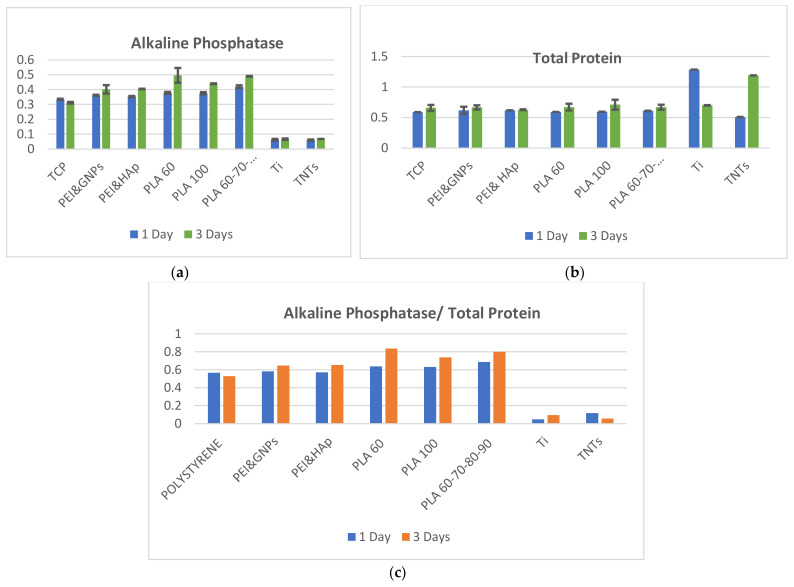
Marker levels expressed in cells when seeded on different substrates, after 1- and 3-days of incubation: (**a**) Alkaline Phosphatase (**b**) Total Protein and (**c**) Alkaline Phosphatase/Total Protein.

In the diagrams in Figure 8, one may see the ALP, TP and ALP/TP ratios, expressed in cells after one and three days of incubation on all tested substrates. More exactly, besides TCP and electrospun substrates, the performance of the following 3D printed scaffolds is analyzed: PLA 60 (polylactic acid with 60 μm pore side length), PLA 100 (polylactic acid with 100 μm pore side length), PLA 60-70-80-90 (multi-layered polylactic acid with 60-70-80-90 μm pore side length). In Figure 8a, it may be observed that the highest ALP levels are expressed in cells on PLA; these overpasses even the ALP in cells on the control material (TCP), which indicates that the 3D printed PLA is appropriate for osteoblasts differentiation. On the other hand, difference between polymeric materials and metallic substrates is extremely pronounced. As observed, all polymeric materials induce positive feedback in cells, with 30% to 50% increase in expressed ALP compared to the metallic substrates. In between all substrates, PLA with 60 μm pore side length promotes ALP production in cells, especially after 3 days of incubation. However, longer incubation periods might show similar results on all pores side lengths, since it was reported that osteoblast lines indicated similar activity on several pore diameters [51]. In Figure 8b it may be seen that the TP level expressed in cells on polymeric materials is relatively constant for both one and three days of incubation. No significant differences are detected in ALP and TP levels when cells are seeded on 60 μm pore side length PLA compared to gradient PLA (60-70-80-90 μm pore dimeters). Increased values were detected for all PLA substrates after three days of incubation compared to the first day. Finally, the diagram in Figure 8c gives a more complete icon on cells response when in contact with the investigated substrates. Higher the ALP/TP, the better since it indicates that strong cell adhesion to substrate does not limit cells development and differentiation. In vivo, osteoblasts’ role is to strongly interconnect and form bone tissue. In vitro, however, they need to adhere to an underneath platform that allows appropriate cytoskeleton expansion, and consequently enables proliferation.

Analyzing the diagram in Figure 8c we conclude that metallic substrates do not promote osteoblasts proliferation. Values of ALP/TP are so low that they are almost undistinguishable after 3 days of cells incubation, when compared to polymeric materials. Polyetherimide composites show similar results independently on the type of reinforcement (GNPs or HAp) and values are comparable to those expressed in the case of TCP. The highest value of ALP/TP is expressed in cells after three days of incubation on 60 μm pore side length PLA.

**Figure 9:**
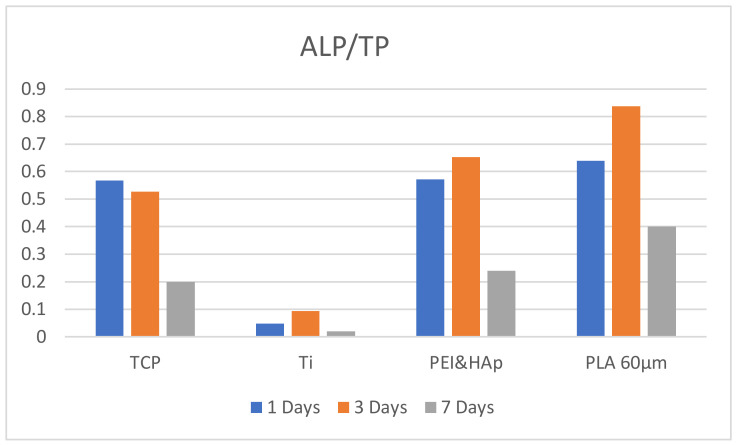
Ratios of Alkaline Phosphatase in osteoblasts seeded for 1, 3 and 7 days of incubation, on the following materials: Tissue culture polystyrene (TCP), Titanium (Ti), Hydroxyapatite reinforced Polyetherimide (PEI&HAp) and polylactic acid with 60 μm pore side length (PLA 60 μm).

One substrate with superior mechanical properties was chosen in each material category, hydroxyapatite reinforced polyetherimide and polylactic acid with 60 μm pore side length, respectively, to perform the analysis of the ALP and TP levels after 7 days of incubation (Figure 9). As observed, the ALP/TP ratios decreases after 7 days of incubation on all substrates, which is normal. The confocal staining presented in the section below demonstrates that the cells populate the materials and consume the migration area; after a while, this leads to the restriction of their development, which is much more evident in the seventh day of incubation for the flat substrates (polystyrene and titanium), where the 3D volume is not available for cells migration. Consequently, cells remodulate their internal processes and delay the multiplication process in order to allow longer viability to the population. The tendency remains constant, showing that the 3D printed scaffolds offer a preferred microenvironment compared to the other substrates.

### 3.4. Cells Staining on Substrates

**Figure 10:**
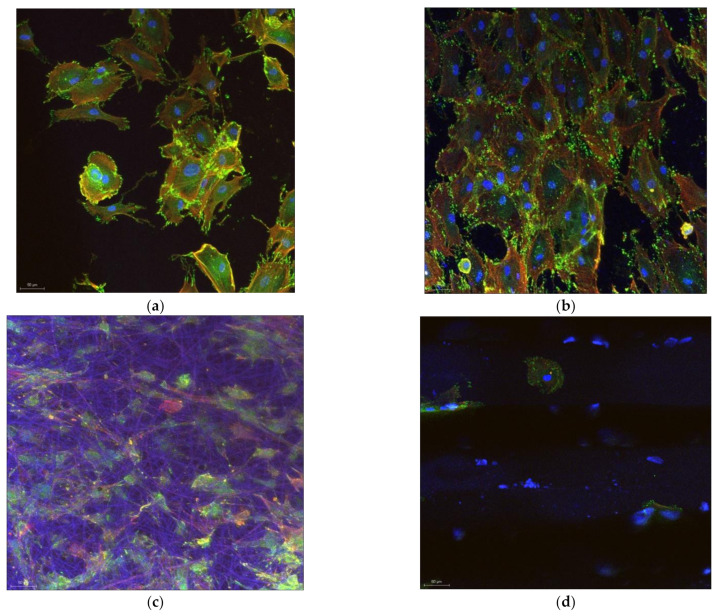
Confocal images of osteoblasts on the substrates after 1 days of incubation on: (**a**) Polystyrene; (**b**) Titanium; (**c**) Electrospun scaffolds and (**d**) 3D printed scaffolds.

Representative confocal images of cells on the various tested substrates can be seen in Figure 10. It must be noted that no differences were found between cells morphology when seeded on the same substrate category e.g., (GNPs reinforced PEI compared to HAp reinforced PEI); more precisely, the reinforcement type in the case of electrospun materials or the diameter of pores on 3D printed scaffolds did not affect the cells morphology or proliferation. The morphology of cells looks almost similar after one day of incubation, when they are seeded on polystyrene (Figure 10a) and on titanium substrates (Figure 10b). Flat substrates visibly promote cells proliferation within the first hours of incubation. However, cells on titanium are less developed and less interconnected compared to those on polystyrene.

3D scaffolds are affected by the dye solution and consequently they are colored in blue. Τhis allows to better observe the 3D structure of the scaffold and how cells migrate in the structure. Electrospun materials induce rapid multiplication, as observed in Figure 10c; cells can be seen in a large number in the whole structure and on different levels of the scaffold. The elastic nature of the electrospun material permits the cells to push the fiber apart and interconnect with other cells. The 3D nature of the electrospun network provides an increased expansion area comparable to flat substrates. In the case of 3D printed scaffolds (Figure 10d), which are robust compared to the electrospun substrates, other rules dominate the cell dynamics (development and migration). After 1 day of incubation, cells cannot be well visualized on the 3D printed scaffolds. Instead, some spheroidal prominences may be distinguished on the fiber surface, which may be salt depositions from the medium. It was concluded that one day of incubation was not sufficient time to assure visible development of cells on 3D printed materials; cells were seeded and further incubated for 7 days.

**Figure 11:**
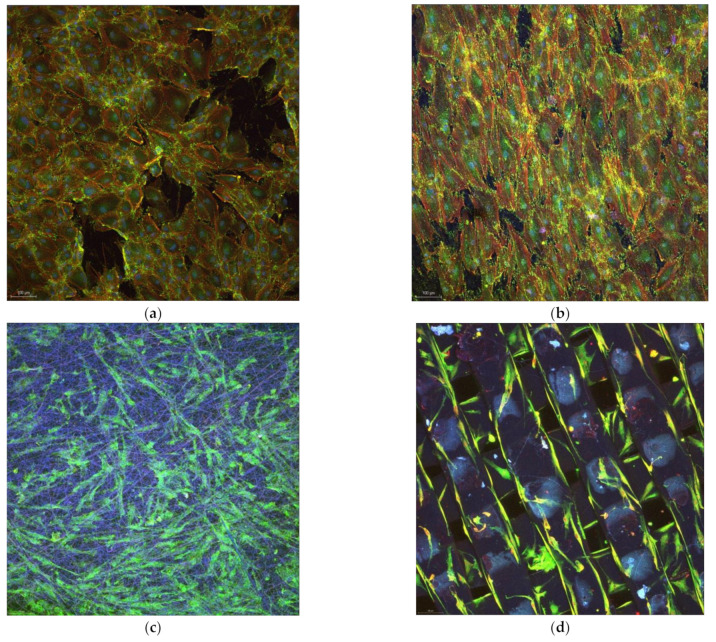
Confocal images of osteoblasts on the substrates after 7 days of incubation: (**a**) Polystyrene; (**b**) Titanium; (**c**) Electrospun; and (**d**) 3D printed.

After 7 days of incubation, cells cover almost the entire surface of the polystyrene substrate (Figure 11a). They are strongly interconnected and form a dense layer with very small, uncovered areas of the substrate. On titanium, cells look spatially restricted; their morphology adapt to the microenvironment and become more needle-shaped to save space. Cells on electrospun scaffold, on the other hand, spread differently penetrating the material and occupying the multi-level space within the scaffold. Although not so prominent as on polystyrene, cells on the 3D electrospun scaffold (Figure 11c) may be seen everywhere on the analyzed area, at the surface and between the underneath fibers. Further on, after 7 days of incubation it may be observed how osteoblasts cover the fibrous area of 3D printed scaffolds (Figure 11d). The robust structure offers a synthetic skeleton-like network which induces positive biosignaling in cells and enables migration. Comparing to the electrospun fibers, the 3D printed ones determine a unidirectional orientation of cells. It may be observed how they develop mainly along the fibers. Finally, all plastic substrates promote proliferation of cells in different modes. Cells on flat polystyrene develop rapidly in a 2D formula, while 3D scaffolds enable proliferation on different levels and directions. In the case of electrospun substrates, cells scan the 3D structure, and it may be seen how they interconnect with cells on a different level within the scaffold. On the 3D printed scaffolds, cells are distributed mainly unidirectionally along the fibers’ length; however, images inside the scaffold cannot be acquisitioned at this point. The two main causes that determine a different behavior of cells on the electrospun scaffolds compared to the 3D printed scaffolds are the nano vs. macro fibers and low vs. high elasticity modulus.

## 4. Discussion

Manipulation of a patient’s biological material in the laboratory is one of the main concerns related to the personalized medicine goal that is intensively promoted nowadays worldwide. Stem cells can be isolated from patients’ bone marrow and differentiated in osteoblasts for development of orthopedic personal cell loaded 3D platforms. Depending on the application, e.g., small bone defects repair with a 3D scaffold or functionalization of an implant surface with a multi-functional coating etc., the solution can be given based on patient’s personal data and needs. In this regard, orthopedic medicine is a convenient specialty with great perspectives in personalized medicine because it gives access to the bone marrow and stem cells, which can be further differentiated into several cell types and returned to the patient. Thus, by combining a patient’s own biological tissue with synthetic materials, scientists can deliver a complex hybrid system composed of a patient’s cells and a synthetic scaffold. To select an ideal platform for a specific application, several lab studies with primary cells extracted from a patient are required with the purpose of standardizing the method. Until now, there is no gold standard to quantify the extent to which substrate characteristics affect cells behavior. Cell populations are receptive to chemical, mechanical, and structural changes but no algorithm is available to describe these processes in a single model. The outcome of comparative biocompatibility studies is essential and will clarify some aspects related to the substrate’s most important characteristics that enable rapid development of bone cells on synthetic tissues.

Within the present investigation, bone marrow was isolated during orthopedic surgery from several patients with the intention of developing a protocol for 3D scaffolds testing with primary cells. The manufactured scaffolds were made of plastics that are commonly accepted as biocompatible; it has been assured that the surface chemistry did not negatively impact cells behavior. Other properties such as the Young’s modulus and the surface texture of the material influenced the quality of interaction between cell population and the underneath substrate. Features of the analyzed scaffolds are given in Table 1.

**Figure 12:**
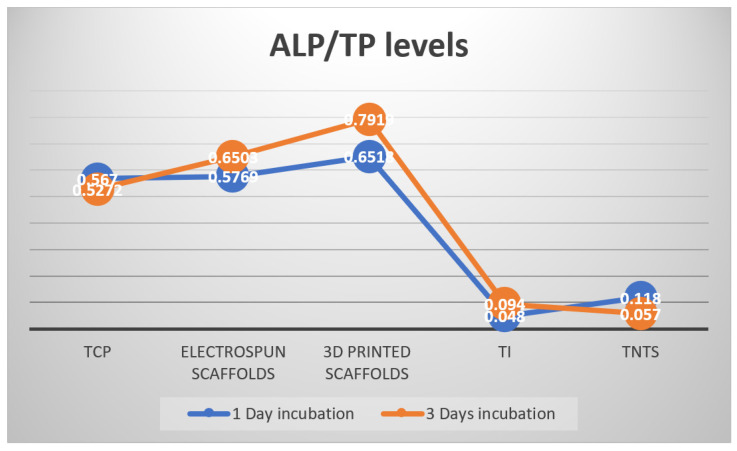
ALP/TP levels measured in osteoblasts and 1 and 3 days of incubation while seeded on the following substrates: TCP (Tissue Culture Polystyrene), Electrospun Scaffolds, 3D printed Scaffolds, Titanium (Ti) andTitania Nanotubes (TNTs).

In previous investigations, expression levels in human osteoblasts increased only on the stiffer surfaces (310 MPa) [53]. Higher matrix moduli in 2D and 3D structures have generally been found to promote osteogenesis. Moreover, it has been recently reported that: (i) osteoblasts migration is enhanced on 3D scaffolds compared to flat surfaces due to higher area provided for cells to develop; (ii) osteoblasts proliferation is migration-dependent and is higher on fibrous materials; (iii) osteoblasts adhesion is improved on materials with higher elasticity modulus and (iv) cells differentiation from MSCs to osteoblasts is adhesion-dependent and is enhanced on stiffer substrates with properties similar to the natural bone tissue [54]. The ratio between the alkaline phosphatase and total protein (ALP/TP) is a good indicator of the balance between the above-mentioned processes. A higher ALP/TP ratio means appropriate migration, proliferation, and adhesion of osteoblasts to a substrate. To depict results in the present investigation, representative values of ALP/TP were selected for each material category (TCP, titanium, TNTs, 3D electrospun scaffolds, and 3D printed scaffolds) and are plotted in Figure 12.

Comparing to the other substrates, 3D printed PLA scaffolds present a micrometer level scaled, highly organized structure, and robustness. All these are advantages that make more suitable the material for new osseous tissue formation within the scaffold. As observed in Figure 12, they induce the best feedback in osteoblasts compared to all the other materials, expressing the highest ALP/TP value.

As previously mentioned, metallic substrates do not promote osteoblasts proliferation. Values of ALP/TP are extremely low after both 1 and 3 days of cells incubation, when compared to the thermoplastic materials (electrospun and 3D printed scaffolds). The ATP/TP values in cells on electrospun substrates are comparable to those expressed in TCP. However, a higher value of ALP/TP is expressed in osteoblasts after three days of incubation on the 3D printed substrates. Analyzing the material features one by one, one may comprehend how they influence the dynamics of osteoblasts population:

1. The control material: TCP has a flat surface and promotes osteoblasts development in a 2D formula; through its plastic nature, TCP does not restrict cells development. Its mechanical properties are intermediate between those of metallic substrates (in the order of GPa) and the 3D printed scaffolds (hundreds of MPa). TCP does not offer a large contact area because of its 2D nature, which makes it restrictive for tissue formation after longer periods of incubation.
2. The metallic substrates: Titanium and TNTs, respectively, are 2D and restrict osteoblasts development because of their chemo-physical nature and their surface texture. The nanometer scale of TNTs does not improve the situation but, on the contrary, determines strong anchorage and swallowing of cells in the substrates, which in turn makes migration difficult and therefore limits proliferation.
3. The 3D electrospinning scaffolds: They are random, and nano-level organized. They have a highly biomimetic structure and a lower elasticity modulus compared to the 3D printed scaffolds. Their performance as orthopedic biomaterial is placed somewhere between TCP and 3D printed scaffolds. In practice, their application can be directed towards surgical practices of the suture or the development of synthetic skin, rather than replacing bone parts. Because of malleability inside the scaffold, cells can push the fibers apart and populate the scaffold within hours. Electrospun scaffolds can be rather associated to dermal than to osseous applications. Overall, it may be declared that electrospun nano-fibers network guide cells towards constructing 3D tissue within the scaffold, which is the main advantage of this type of material.
4. 3D printed scaffolds: They are highly organized robust structures made of microfibers. The micro-level is not perceived by the biologic component as a 3D structure. The reason is that osteoblasts’ length may reach 10 μm, and therefore they are smaller in dimensions than the fibers and the pores of the substrate. Despite that, cells populate the fiber with a tendency to develop unidirectionally along the fibers. The mechanical property of the material makes it suitable for the biosignaling of bone cells as for instance osteoblasts. On these types of scaffolds, osteoblasts express the highest ALP/TP value. The 3D printed scaffold biomimic well the internal structure of osseous tissue and promote in vitro tissue formation.

The above results show that the 3D printed substrates are superior with respect to their perspectives in orthopedic implantology. This is because of their overall properties which are: balanced mechanical properties, biomimetic features such as real bone, highly organized structure, and biodegradability. The biomimetic features of the manufactured scaffolds enable osteogenesis by contributing to bone cells’ differentiation and appropriate development reflected through a high ALP/TP ratio. As stated above, Alkaline Phosphatase is an important component in hard tissue formation, highly expressed in mineralized tissue cells and the most widely recognized biochemical marker for osteoblast activity. The scaffolding material (PLA), together with the growth factors that have been used to induce osteogenesis, are determining the pronounced differentiation into osteoblasts. Comparing the TCP control material and the PLA scaffolds, we observe an improvement in the case of the PLA. Material composition, porosity that allows the uniform flow of the culture medium, and the good mechanical properties of the 3D printed PLA scaffolds are the main factors that contribute to the osteogenic differentiation. The function of these scaffolds is to augment bone regeneration via osteoinduction of the seeded progenitor cells as well as osteoconduction, which has been achieved by interplaying these factors such as surface and bulk properties.

## 5. Conclusions

The present research was based on a cooperation and exchange of practices between personnel working in the medical area, biomedical researchers, and engineers, to achieve the creation of an efficient circuit that allows the harvesting of bone tissue during orthopedic surgery, the isolation of MSCs and their differentiation into osteoblasts to perform biocompatibility studies on 3D scaffolds elaborated by novel technologies (electrospinning and 3D printing). The most time-consuming part of this circuit model has been the one associated with the approval of the research by the ethical committee (around 6 months). This consisted in training the medical staff for the tissue harvesting procedure and for obtaining patients’ approval. The ethics committee has approved the procedure under the following conditions: (1) the patients will be accordingly informed; (2) only residual tissue will be harvested and used for laboratory research and (3) isolated cells are not going to be used for cloning. The extraction and transportation protocol has been established, while the maximum measures to avoid contamination of the samples were considered and consisted in steps inside the surgery room (ethanol sterilization of the tubes before and after opening them, parafilm and aluminum foil isolation) and from the surgery room to the lab (immediate transportation to the lab in a well isolated thermobox). Half of the residual bone samples did not result in cell growth (10 out of 19 samples were successful). The unsuccess rate can be attributed to the undocumented associated pathology of donors. Positive samples resulted in successful MSCs cultures that were further differentiated into osteoblasts through the appropriate culture medium, to perform a study of biocompatibility on different 3D scaffolds. ALP and TP levels in osteoblasts seeded on different substrates were quantified after 1, 3 and 7 days of incubation and cells were stained to observe the development of their cytoskeleton on and into the materials.

Three types of electrospun scaffolds (Supramolecular, GNPs reinforced polyetherimide, HAp reinforced polyetherimide) and three types of 3D printed scaffolds (single or multi-layered Polylactic Acid with square shaped pores having side lengths 60 or 80 or 60-70-80-90 μm) were tested against tissue culture polystyrene, titanium, and titanium dioxide nanotubes. In the case of the electrospun materials, results showed that almost similar ALP levels are expressed in osteoblasts on all of them, with the light increasing for the two polyetherimide composites compared to the supramolecular. ALP levels are higher in osteoblasts on electropsun materials compared to TCP and titanium substrates, which makes these substrates superior, indicating appropriate proliferation and differentiation. Further on, the two polyetherimide composites promote the production of appropriate TP amount in cells. Electrospun polyetherimide scaffolds induce maturation of cells, differentiation, and convenient adhesion. No significant differences are observed in between GNPs reinforced PEI and HAp reinforced PEI. Supramolecular also induces high ALP activity in cells; however, the values decrease after 3 days of culture, which indicates that the material should be tested for longer periods to decide whether it is or not toxic and appropriate for in vitro tissue formation. The 3D printed PLA scaffolds induced significantly improved behavior in the primary osteoblasts compared to all the other tested substrates (tissue culture polystyrene, metals, electrospun scaffolds). This is because the 3D printed scaffold biomimic the spongy bone structure and enable bio-recognition. Higher ALP amounts were detected and resulted in increased ALP/TP ratios, indicating a faster biointegration rate due to adjusted adhesion associated to a good proliferation rate. The superiority of the printed scaffolds can be attributed to their structure and overall features that guide cells migration, while they determine a unidirectional distribution of cells along the fiber length. The increased incubation times of cells (7 days) resulted in a decrease of the ALP/TP ratios, which is associated with a reduced space for cells migration. Larger samples are needed for extended incubation times. Staining of the cells on all substrates confirmed the biomarker analysis and the interpretation.

An important finding is that a 30% to 50% improvement of expressed ALP can be observed in cells on plastic substrates compared to metallic ones. This proves that the addition of plastic coatings on metallic implants is highly demanded. Titanium generally restricts cells development, which has been underlined through the present investigation.

This study shows that PLA 3D printed scaffolds are promising grafts for bone repair in the case of small trauma, while 3D electrospun scaffolds are more appropriate as coatings on orthopedic implants, to assure rapid and efficient osseointegration. Future investigations aim to improve the mechanical and overall properties of the manufactured scaffolds and to evaluate their biocompatibility by means of primary osteoblast cells, for longer incubation periods and under different microenvironmental conditions. Complex investigations of biomimetic scaffolds for orthopedic applications are needed to finally deliver personalized cell carrying autografts to patients. Furthermore, by combining several 3D manufacturing strategies, the artificial vascularization of the constructed scaffolds could be achieved for the simultaneous reconstruction of both osseous and endothelial tissues [55].

## Figures and Tables

**Table 1:** Bulk and surface properties of plastic substrates.

Material Type		Young’s Modulus [MPa]	Surface Type and Level (nm/μm)	Organization
**Tissue culture Polystyrene (TCP)** [52]		3000	Compact	-
**Supramolecular (SP)** [42]		350	Fibrous-nm	Random
**Electrospun materials** [43]	GNPs reinforced PEI	53	Fibrous-nm	Random
	HAp reinforced PEI	130	Fibrous-nm	Random
**3D printed scaffolds** [44]	PLA 60 μm	200	Fibrous-μm	Highly organized
	PLA 100 μm	170	Fibrous-μm	Highly organized
	PLA60-70-80-90μm	190	Fibrous-μm	Highly organized

## Data Availability

Not applicable.
